# Exploring the association between the degree of pyuria and urinary tract infections

**DOI:** 10.1128/spectrum.02015-24

**Published:** 2025-03-05

**Authors:** Hsin Tien, Katherine Bond, Wei Hong, Katie Cronin, Eddie Chan

**Affiliations:** 1 Department of General Medicine, Royal Melbourne Hospital, Parkville, Victoria, Australia; 2 Department of Infectious Diseases, Royal Melbourne Hospital, Parkville, Victoria, Australia; 3 Department of Microbiology, Royal Melbourne Hospital, Parkville, Victoria, Australia; 4 Personalised Oncology Division, Walter and Eliza Hall Institute of Medical Research, Parkville, Victoria, Australia; Hartford Hospital, Hartford, Connecticut, USA

**Keywords:** urinary tract infection, pyuria, antimicrobial stewardship

## Abstract

We aim to define the optimal white cell count threshold that correlates with a clinically significant urinary tract infection, as there is insufficient data exploring this in the adult population. We conducted a retrospective cohort study at the Royal Melbourne Hospital analyzing urine samples collected over 6 months in 2022. Urinary tract infection (UTI) was defined as the presence of symptoms (dysuria, urgency, frequency, flank pain, or loin to groin pain) and isolation of a uropathogen with colony counts greater than 10^7^ CFU/L. The relationship between urinary white cell count, growth of uropathogen, and likelihood of UTI was estimated using locally weighted scatterplot smoothing. Of the 6,328 samples included, at a urinary white cell count of less than 10 per microliter, 38% grew a microorganism, while 7% of the total grew a uropathogen. For our sub-analysis part C, at the same WBC count, 2% of samples fulfilled our criteria for UTI. The optimal WBC range for identifying a UTI was 30–50 WBC/µL, demonstrating the most pragmatic balance of sensitivity (92.3%–94.9%, 95% CI 85.9–98.1) and specificity (41.6–47.2, 95% CI 38.6%–50.3%) for a UTI. A lower-than-expected number of UTIs were confirmed in our study, likely due to inappropriate indications for culture collection and the types of urine specimens collected. However, the optimal white cell cutoff for identifying a UTI was higher than the defined pyuria cutoff of 10 WBC/µL. Utilizing a higher urinary WBC cutoff could improve urine culture processing protocols.

**IMPORTANCE:**

The importance of this study is that it explores the optimal range of white cell count that defines a clinically significant urinary tract infection. The traditional cutoff of more than 10 white blood cells per microliter is often used; however, we now recognize that using this cutoff may not accurately represent true urinary infections, especially in certain populations such as young women. The uncertainty in the optimal white cell count cutoff has widespread implications. For the clinician, it may lead to unnecessary prescribing of antibiotics for patients who do not have a Urinary tract infection, such as asymptomatic bacteriuria. For the microbiology laboratory, it may lead to unnecessary workup and culture of urine samples that are not clinically relevant. Many studies to date have attempted to define an optimal value for urinary white cell count; however, not many have incorporated relevant clinical data, which this study has.

## INTRODUCTION

The urine sample is among the most common diagnostic specimen types received by the routine microbiology laboratory (1). A urinary tract infection (UTI) diagnosis is made on the basis of both clinical symptoms (e.g., dysuria, frequency, urgency, or costovertebral angle tenderness) and laboratory parameters, such as pyuria, as well as a positive urine culture with a bacterial growth of ≥10^5^ CFU/mL (2). However, culturing urine specimens is both time and labor-intensive, with up to 60%–70% of cultures eventually returning negative for growth (3, 4). Thus, there is a need to implement additional diagnostic algorithms, such as the urine white cell count, for the laboratory to avoid unnecessarily culturing a urine specimen from patients with a lower likelihood of true clinical UTI.

Pyuria, or the presence of white blood cells (WBCs) in urine, has traditionally been defined as more than 10 WBC/µL (5, 6) or five or more WBC per high power field (7–9). This definition is based on Mabeck’s work (10) in the 1950s, which demonstrated that a leucocyte excretion rate of >400,000/hour had a positive association with bacteriuria and UTI symptoms. Notably, a leucocyte excretion rate of >400,000/hour equated to >10 leukocytes/mm^3^ in unspun urine samples via cell count analysis on hematocytometers (5). Various studies have consistently shown that a clinically significant UTI is almost always accompanied by pyuria (5, 10–14). However, up to 5% of young women and up to 90% of elderly institutionalized patients also fulfill the same criteria for pyuria in the absence of symptoms (15, 16). This suggests that the specificity for pyuria as a sole marker in distinguishing asymptomatic from symptomatic infections is low (17, 18). In addition, clinicians commonly prescribe unnecessary antimicrobial therapy based on asymptomatic bacteriuria (19) due to an overreliance on “red flag” parameters, such as pyuria, positive nitrites, and gram-negative bacteriuria (20). This not only increases the risk of antimicrobial resistance but incurs greater costs in terms of screening and treatment for both the healthcare system and patients (2, 21).

There is limited literature examining suitable urine WBC count thresholds that correspond to the likelihood of a clinical UTI diagnosis in an adult population (8, 18). Various studies based on flow cytometer analyzers have attempted to define an optimal cutoff value for WBC that corresponds to either bacteriuria or symptomatic UTI, though most do not include clinical symptoms (22). Of note, the cutoffs vary greatly, ranging from 20 WBC/µL to more than 100 WBC/µL (4, 23–27). This is due to broad heterogeneity in patient cohorts, including demographics and co-morbidities, the different prevalence of UTI, and the utility of specific urine analyzers (28).

The UK Standards for Microbiological Investigations guidelines define significant pyuria as greater than 10 WBC/µL in line with Mabeck’s definition above; however, they acknowledge that a higher level of greater than 100 WBC/µL could be a more appropriate cell count threshold (12). This appears to be a pragmatic approach to improve the specificity of the UTI diagnosis. In the Australian context, the Royal College of Pathologists of Australasia manual for the use and interpretation of pathology tests defines significant pyuria by recommending greater than 40 WBC/µL as suggestive of infection (29). Given the challenges in seeking a uniform cutoff, local Australian laboratories often use their own WBC thresholds based on their patient population to determine whether further identification and susceptibility testing of urinary isolates is warranted. This, in turn, influences diagnostic reporting approaches, which are designed to support accurate interpretation of results, as well as antimicrobial stewardship practices. Utilizing thresholds that prioritize sensitivity over specificity increases the work of the microbiology laboratory on isolates that are not clinically relevant and increases the risk of clinicians unnecessarily treating asymptomatic bacteriuria. As urine is a high-frequency laboratory test type, an algorithmic approach to microbiological workflow is necessary for efficient utilization of labor and reagent resources.

Our study was designed to better understand the relationship between urine white cell count, bacteriuria, and a clinically significant UTI. Our main objectives were to ascertain the probability of (i) isolating an organism at varying urine white cell counts; (ii) isolating a uropathogen (see definition in Materials and Methods); and (iii) a clinical UTI based on laboratory and clinical data.

## MATERIALS AND METHODS

This was a retrospective, single-center study conducted at the Royal Melbourne Hospital (RMH), Victoria, Australia, evaluating urine specimens collected from patients between April and September 2022. Specimen data were retrieved from the RMH laboratory information system Auslab (Victoria, Australia), while clinical information was retrieved from the electronic medical record system (EPIC, Wisconsin, USA). Laboratory information collected included urine white cell count, organism(s) isolated, urine culture plate bacterial colony count, and type of urine sample (e.g., midstream, in-out catheter/straight catheter, or clean catch). Urine white cell count indices were derived from testing on the automated Beckman Coulter Iris iQ200 urine analyzer (California, USA). Urine culture was performed on recommended microbiological split horse blood and MacConkey agar medium.

**Fig 1:**
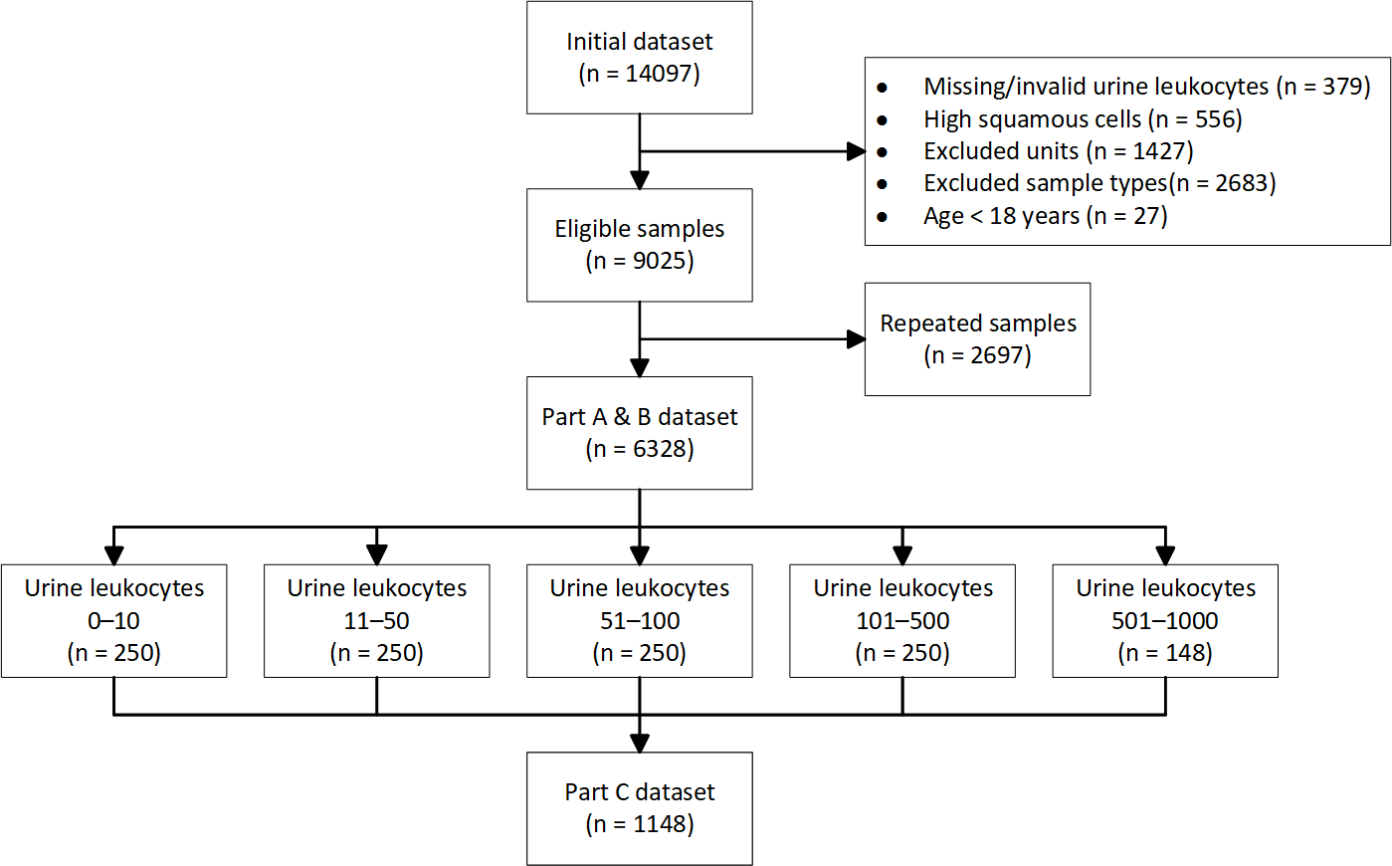
Summary of the selection process of urine microscopy, culture, and sensitivity samples.

To ensure the generalizability of patient results, specimens taken from the Intensive Care Unit, Hematology, and Oncology wards were excluded from analysis, as these patients are heterogeneous in their immunosuppressed state and therefore not reflective of our target patient population group (Fig. 1). Other exclusion criteria include specimens collected from patients under the age of 18, subsequent urine specimens from the same patient in the testing period of 6 months, and less optimal or non-sterile specimen types, such as first void urine, urine bag, indwelling catheter, ileal conduit, and nephrostomy tube. In addition, we excluded urine samples that had high squamous epithelial cells (three or more) on urinalysis.

The analysis was divided into three parts (Fig. 1). For parts A and B, all samples that met inclusion criteria in the testing period were analyzed. In part A, we measured the probability of unspecified organism growth in urine at different urine white cell count values. In part B, we measured the probability of isolation of a uropathogen (Table S1) at different urine white cell values, with a definition of uropathogen consistent with recent literature (8, 26). As analysis of the presence/absence of a uropathogen is a binary outcome, organisms that are only occasionally implicated as a causative organism in a UTI (e.g., *Enterococcus faecalis* and *Pseudomonas aeruginosa*) were classified as uropathogens. Other organisms that are rarely attributed to a clinical UTI were classified as non-uropathogens.

For part C, we measured the percentage of samples that met the criteria for a clinical UTI. Five test groups were arbitrarily created based on different urine white cell count values, as follows: less than 10, 10–50, 51–100, 101–500, and 501–1,000 WBC/µL. Each test group comprised the first up to 250 consecutive specimens by date from our data set to create a subset of up to 1,250 urine samples. We evaluated if samples were consistent with a clinical UTI, based on an algorithm (Table S2) where we defined clinical UTI as present if each of these criteria were fulfilled: presence of at least one UTI symptom, isolation of a uropathogen with plate colony counts greater than >10^7^ CFU/L, and either a single or predominant organism. This is consistent with the laboratory definition of a UTI as outlined in guidelines, such as the UK standards for microbiology investigations—“Investigation of Urine” and Infectious Diseases Society of America’s “Clinical Practice Guideline for the Management of Asymptomatic Bacteriuria” (2, 12). We did not include non-specific symptoms, such as lower abdominal pain or fever. Samples were cross-referenced with our electronic hospital medical record to look for the presence of UTI symptoms present at the time of urine collection (i.e., dysuria, urgency, frequency, flank pain, or loin to groin pain). Specimen records from our laboratory information system were interrogated to ascertain details on isolate speciation, the number of different organisms isolated, and plate bacterial colony counts.

The relationship between urinary white cell counts and each of the three outcomes (probability of a microorganism, uropathogen, and clinical UTI) was non-parametrically estimated using locally weighted scatterplot smoothing (LOWESS) with a bandwidth of 0.8. Means and confidence intervals for each LOWESS curve were estimated using bootstrapping with 10,000 replicates. A receiver operating characteristic curve analysis was also conducted to calculate Youden’s index, which provides a reference for the optimal WBC cutoff. All analyses were conducted using Stata version 16.1 and R version 4.3.2.

## RESULTS

A total of 14,097 samples were screened, of which 6,328 samples were included in parts A and B, while 1,148 were included in part C (Fig. 1). Table 1 provides epidemiological data for our cohort.

**TABLE 1:** Epidemiology table—patient characteristics

	Years
Age	
Mean	61.0
Range	18.0–101.7
Sex	*N* (%)
Female	3,133 (49.5)
Male	3,190 (50.4)
Other	5 (0.1)
Location	*N* (%)
Emergency	3,278 (51.8)
Inpatient	2,287 (36.1)
Outpatient	763 (12.1)

**Fig 2:**
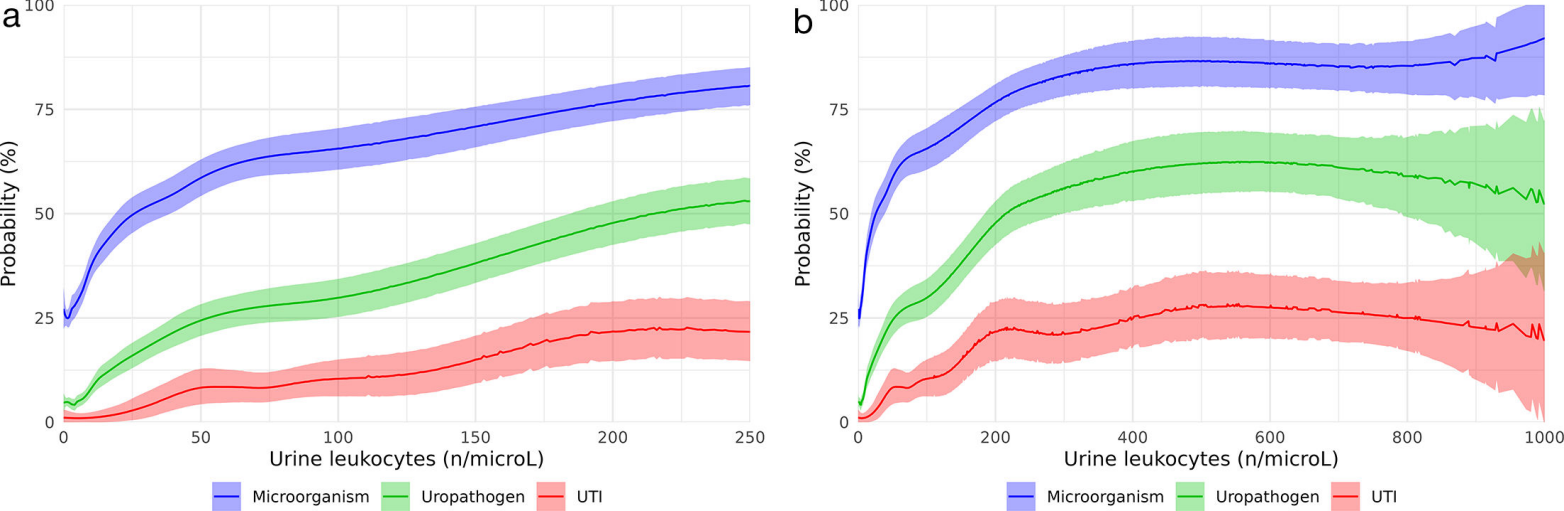
The percentage of samples growing a microorganism, a uropathogen, and fitting our UTI criteria, plotted against leucocyte count (from 0 to 1,000 WBC/µL). Panel a shows leucocyte count between 0 to 250 WBC/µL and Panel b shows leucocyte count between 0 to 1000 WBC/µL.

Figure 2 shows the proportion of samples growing (i) any microorganism, (ii) any uropathogen, and (iii) those that fit our criteria for a UTI at different urine white cell counts. We found that at 10 WBC/µL, 38% of samples isolated a microorganism, with 7% of the total samples being identified as a uropathogen. At a urine white cell count of 50 WBC/µL, 58% grew a microorganism, with a 25% isolation rate of a uropathogen. At 100 WBC/µL, this increased to 66% for the isolation of any organism and 30% for a uropathogen. Figure 2 also shows the number of specimens that met clinical criteria for a UTI at various WBC counts in part C. This was 3% at 20 WBC/µL, 8% at 50 WBC/µL, and 10% at 100 WBC/µL. At a WBC count of greater than or equal to 500 WBC/µL, 28% met our criteria for a UTI (Fig. 2b).

**Fig 3:**
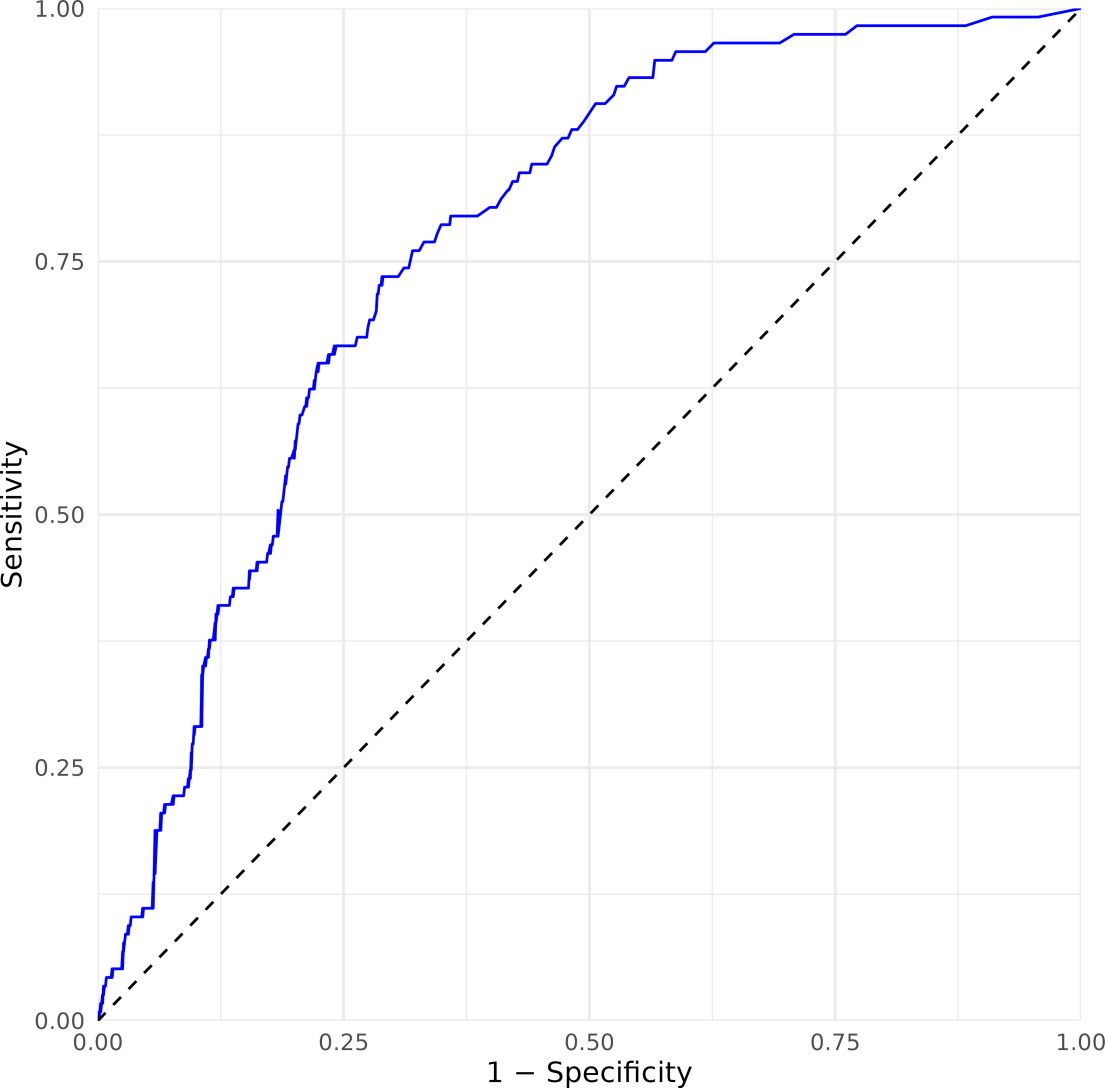
Receiver operating characteristic curve of urine leukocytes as a predictor of UTI. Based on maximizing Youden’s index, the optimal WBC cutoff is 110 WBC/µL (sensitivity 0.74 and specificity 0.71).

Sensitivity, specificity, positive predictive value, and negative predictive value for the diagnosis of a UTI at various cutoffs of urine white cell count are shown in Table 2. A receiver operating characteristic curve has also been included (Fig. 3), which has determined the ideal urine white cell count cutoff as 110 WBC/µL.

**TABLE 2:** Part C—sensitivity, specificity, positive predictive value, and negative predictive value at different white cell counts for diagnosing urinary tract infection^*a*^

Cutoff, leukocytes/µL	Sensitivity, % (95% CI)	Specificity, % (95% CI)	PPV, % (95% CI)	NPV, % (95% CI)
10	98.3 (94.0–99.8)	22.8 (20.3–25.5)	12.6 (10.5–15.0)	99.2 (97.0–99.9)
20	96.6 (91.5–99.1)	35.5 (32.6–38.5)	14.5 (12.1–17.2)	98.9 (97.3–99.7)
30	94.9 (89.2–98.1)	41.6 (38.6–44.7)	15.6 (13.0–18.4)	98.6 (97.0–99.5)
40	93.2 (87.0–97.0)	44.5 (41.5–47.6)	16.0 (13.3–19.0)	98.3 (96.7–99.3)
50	92.3 (85.9–96.4)	47.2 (44.2–50.3)	16.6 (13.8–19.6)	98.2 (96.6–99.2)
75	80.3 (72.0–87.1)	59.8 (56.8–62.9)	18.5 (15.2–22.2)	96.4 (94.7–97.7)
100	73.5 (64.5–81.2)	69.4 (66.5–72.2)	21.4 (17.5–25.8)	95.9 (94.2–97.2)
150	66.7 (57.4–75.1)	75.8 (73.0–78.3)	23.8 (19.3–28.8)	95.2 (93.6–96.6)
200	60.7 (51.2–69.6)	79.0 (76.3–81.4)	24.7 (19.8–30.1)	94.7 (92.9–96.1)
300	43.6 (34.4–53.1)	84.6 (82.2–86.7)	24.3 (18.6–30.7)	93.0 (91.1–94.5)

^*a*^ CI, confidence interval; PPV, positive predictive value; and NPV, negative predictive value.

## DISCUSSION

In this study, we analyzed a cohort of tertiary adult hospital patients to identify the optimal white blood cell cutoff that best correlates with a clinically significant urinary tract infection. The correlation with clinical symptoms, crucial to the diagnosis of a UTI, allows the exclusion of samples consistent with asymptomatic bacteriuria, which do not require antimicrobial treatment (2). As the urine specimen is by far the most common microbiological test type ordered by clinicians, there is substantial potential for the administration of unnecessary antimicrobial therapy if inappropriately collected, analyzed, and subsequently reported. This has broad downstream implications not limited to the effects on the host microbiome, propagation of antimicrobial resistance, drug interactions, healthcare-associated medication, and inpatient treatment costs. This theme of judicious test selection is in line with the Choosing Wisely Campaign launched in North America in 2012, which has since been endorsed and adopted by numerous international medical societies, including the Infectious Diseases Society of America, Australasian Society for Infectious Diseases, and The Royal College of Pathologists of Australasia (30–33). One such measure to support good quality antimicrobial prescribing is through the implementation of effective pathology diagnostic and reporting stewardship, such as the microbiology test report output (34, 35).

Although numerous studies have evaluated the use of urine WBC cutoffs, far fewer have incorporated urinary symptoms in their analysis (8, 26, 36–38). This is a clear limitation, given the presence of symptoms is a fundamental component of a UTI diagnosis (2). While having a targeted cutoff should improve laboratory stringency in the workup of urine samples, this has not been reflected in daily laboratory practice. Our study incorporated urinary symptoms into an algorithm to more accurately define the presence of a true clinical UTI. In previous publications, a wide range of WBC cutoffs were defined with variation due to patient age difference, gender, and presence of co-morbidities (8, 26, 36–38). Bilsen et al. (8) found that for older women ≥65 years old, a cutoff of more than 264 WBC/µL was needed to obtain sensitivity and specificity of 88%, whereas Foudraine et al. (26) found that 74 WBC/µL was an optimal cutoff in their tertiary hospital population with a large proportion of immunocompromised patients. Furthermore, while more than half of these studies were limited to the emergency department setting (36–38), our study was more permissive in the inclusion of inpatients, outpatients, and emergency department patients, which would reflect the case mix expected from a routine diagnostic microbiology laboratory.

Through our study, we found that, as expected, the probability of bacteriuria increased with increasing WBC counts. However, using the traditional cutoff for pyuria of greater than 10 WBC/µL was not sufficient for ruling out UTI.

We found that at the non-pyuria cutoff (10 WBC/µL), approximately 40% of samples grew a microorganism, and about 10% grew a uropathogen. Despite this, the probability of a UTI upon a full assessment of patient symptoms and microbiological plate culture data was remote, at 2%. This indicates that bacteriuria is common, and the positive predictive value for a UTI based on the growth of a uropathogen is poor in this urine WBC count range. Bacteriuria in this setting is thus more likely reflective of contaminated urine specimen collection or normal urethral bacterial colonization rather than a true UTI. This is supported by non-uropathogens accounting for 75% of organisms cultured between the range of 0 and 10 urine WBC/µL.

As expected, the likelihood of bacteriuria increased in tandem with a rise in urine leucocyte count. This corresponded with a rise in the probability of a clinical UTI, reaching a maximum of around 28%. This was somewhat surprising as we expected a higher overall probability of UTI, particularly with higher urine white cell counts. One explanation for this was that the indication for urine specimen collection often did not meet our specified symptom criteria for a clinical UTI. For instance, samples were noted to have been collected for asymptomatic patients attending outpatient clinics, such as renal or urology clinics, where there is no record of urinary symptoms, thus lowering the pre-test probability of a UTI. We anticipate that the ease of urine sample collection for culture purposes in a predominantly inpatient hospital as compared to an ambulatory/primary care setting may have contributed to this. Further research should evaluate if rates of urine specimen requests in the primary care setting are better aligned with positive UTI symptoms. In addition, negative urine cultures or low organism colony counts (interpreted as a negative UTI result) are plausible in the situation of a partially treated UTI, although given more than half of our data are from an ED setting (52%), where we assume most presentations are a first presentation of urinary symptoms, we expect this to be a relatively smaller number.

For the laboratorian, the sensitivity of identifying a UTI at a designated urine WBC cutoff is useful to determine the need for further urine isolate workup and culture. In considering the optimal cutoff, many choose cutoffs that maximize Youden’s index to obtain an equally optimized sensitivity and specificity. However, as Schuh et al. point out (36), such strategies are often not clinically useful as they can neither rule in nor rule out a disease. Therefore, for a more balanced approach, it is imperative to identify the pre-test probability of a disease and how much we can afford a misdiagnosis (39). This means that cutoff values should be adjusted to individual diseases and contexts. We opted for a WBC cutoff that resulted in a higher specificity and lower sensitivity for the below reasons. First, our laboratory, generalizable to other routine diagnostic clinical microbiology laboratories, receives a high volume of urine samples. In addition, urine culture is considered a sample type of lesser importance as compared to a specimen of higher clinical impact, such as a blood culture or cerebrospinal fluid sample. A lower sensitivity would therefore entail less unnecessary workup of culture-positive samples not consistent with a clinical UTI syndrome. In exchange, we accept a slightly higher rate of false negatives. In accordance with our national governing laboratory quality standards, we are mandated to keep cultured isolates for a period of time following reporting, and when clinically indicated, the workup of these isolates can be communicated between the clinician and laboratory staff. In these instances, minimal patient harm is expected from a delay to antimicrobial susceptibility testing and subsequent directed antimicrobial therapy.

For our study, the difference between the sensitivity of identifying a UTI at a cutoff of 10 WBC/µL compared to a cutoff of 50 WBC/µL was negligible (98% vs 92%), while the gain in specificity was significant (23% vs 47%). However, the sensitivity dropped to 74% when a cutoff of 100 WBC/µL was applied. We infer that the appropriate urine white cell count cutoff that would indicate the need for progression to urine culture, therefore, should be in the range of 30–50 WBC/µL, demonstrating the most pragmatic balance of sensitivity (92.3%–94.9%, 95% CI 85.9–98.1) and specificity (41.6–47.2, 95% CI 38.6%–50.3%) for a UTI. This, however, should clearly be adjusted to the clinical setting—laboratories that principally service hospital inpatients might opt for a higher urine white cell count threshold compared to the one that caters to primary care clinicians.

The strengths of this publication lie in its sizeable test cohort, the number of urine samples analyzed, and the review of relevant patient clinical history. By providing information on UTI probability as well as sensitivity calculations at different white cell counts, this is relevant to clinicians and laboratorians, both for assessing the need for treatment and for urine specimens that require further laboratory workup (e.g., susceptibility testing). We used widely accepted laboratory criteria to define a UTI. While a UTI is possible with a colony counts less than 10^7^ CFU/L, this is more frequently recognized in healthy young females, which is not the predominant patient cohort in this study. Limitations of this study include it being a single-center study from a tertiary-level adult hospital. There is potential for institution-specific practices in urine specimen collection, which may affect the generalizability of these results. As this is a retrospective study, the quality of the analysis relied heavily on the accuracy of documentation in our electronic medical records, including the indication for urine culture that clinicians request. The simplicity of this study lends itself to comparable studies in different settings, such as in other tertiary-level hospitals, secondary hospitals, and primary care, which would then confirm if our findings are more broadly generalizable. We excluded urine specimens collected from the Intensive Care and Hematology-Oncology wards to ensure our analysis was performed on a group of patients who were not significantly immunocompromised or critically unwell.

Our study demonstrated that the WBC count of a urine sample is predictive of the probability of a UTI, and the optimal range was between 30 and 50 WBC/µL in our cohort, confirming its role as a key threshold for the implementation of laboratory stewardship. Additionally, we identified a substantial number of urine culture requests that were incongruous with a suspected urinary tract infection, which highlights the need to provide ongoing education to health practitioners. With practice change, this may lead to further re-calibration of clinically significant urine white cell count values.

## Data Availability

The data for this study are available from the authors upon reasonable request.

## SUPPLEMENTAL MATERIAL

Supplemental material spectrum.02015-24-s0001.docx Tables S1 and S2.

ASM does not own the copyrights to Supplemental Material that may be linked to, or accessed through, an article. The authors have granted ASM a non-exclusive, world-wide license to publish the Supplemental Material files. Please contact the corresponding author directly for reuse.
