# Supplementary material for: Exploring the association between the degree of pyuria and urinary tract infections
Source: Microbiol Spectr. 2025 Mar 5;13(4):e02015-24. doi: 10.1128/spectrum.02015-24 (PMC11960134; doi:10.1128/spectrum.02015-24)
Supplement: Supplemental material — Tables S1 and S2. [file spectrum.02015-24-s0001.docx]

**Supplemental Material**

| Name | n | % |
| --- | --- | --- |
| Uropathogens: | 1269 | 20.05 |
| Beta-haemolytic Streptococcus | 40 | 0.63 |
| Citrobacter spp. | 27 | 0.43 |
| Corynebacterium urealyticum | 1 | 0.02 |
| Enterobacter spp. | 33 | 0.52 |
| Enterococcus faecalis/faecium | 115 | 1.82 |
| Escherichia spp. | 664 | 10.49 |
| Klebsiella spp. | 126 | 1.99 |
| Proteus spp. | 105 | 1.66 |
| Pseudomonas aeruginosa | 70 | 1.11 |
| Staphylococcus aureus/saprophyticus | 36 | 0.57 |
| Other Enterobacterales | 43 | 0.68 |
| Other miscellaneous uropathogen | 9 | 0.14 |
| Non-uropathogens: | 239 | 3.78 |
| Miscellaneous gram-negative organism | 7 | 0.11 |
| Miscellaneous gram-positive organism | 9 | 0.14 |
| Non-uropathogenic Corynebacterium | 17 | 0.27 |
| Other Pseudomonas | 1 | 0.02 |
| Other Staphylococcus | 94 | 1.49 |
| Other Streptococcus | 37 | 0.58 |
| Yeast | 74 | 1.17 |

**Supplementary Table 1.** Microorganisms included in analysis. Some urine samples had more than one organism. Percentage based on total number of samples analysed in parts A & B, which is 6328.

| Symptoms (dysuria, frequency, urgency, flank or loin to groin pain) | Colony count greater than > 10^7^ CFU/L | Uropathogen | Either a single or predominant organism | Meets criteria for UTI |
| --- | --- | --- | --- | --- |
| Y | Y | Y | Y | Y |
| Y | Y | Y | N | N** |
| Y | Y | N | Y | N |
| Y | N | Y | Y | N |
| N | Y | Y | Y | N |
| Y | Y | N | N | N |
| Y | N | Y | N | N |
| N | Y | Y | N | N |
| Y | N | N | N | N |
| N | Y | N | N | N |
| N | N | Y | N | N |
| N | N | N | Y | N |
| N | N | N | N | N |
| N | N | Y | Y | N |
| N | Y | N | Y | N |
| Y | N | N | Y | N |

**Supplementary Table 2.** Urinary tract infection diagnostic algorithm for this study. Abbreviations: UTI (Urinary tract infection), N = No, Y = Yes.

**unless 2 organisms with equal colony counts then this counted as a UTI (Y).
